# A Feature Tensor-Based Epileptic Detection Model Based on Improved Edge Removal Approach for Directed Brain Networks

**DOI:** 10.3389/fnins.2020.557095

**Published:** 2020-12-21

**Authors:** Chuancheng Song, Youliang Huo, Junkai Ma, Weiwei Ding, Liye Wang, Jiafei Dai, Liya Huang

**Affiliations:** ^1^Bell Honors School, Nanjing University of Posts and Telecommunications, Nanjing, China; ^2^College of Electronic and Optical Engineering, College of Microelectronics, Nanjing University of Posts and Telecommunications, Nanjing, China; ^3^Neurology Department, the General Hospital of Eastern Theater Command, Nanjing, China; ^4^National and Local Joint Engineering Laboratory of RF Integration and Micro-Assembly Technology, Nanjing, China

**Keywords:** short-term EEG data, edge removal, epileptic detection, feature tensor, directed brain network

## Abstract

Electroencephalograph (EEG) plays a significant role in the diagnostics process of epilepsy, but the detection rate is unsatisfactory when the length of interictal EEG signals is relatively short. Although the deliberate attacking theories for undirected brain network based on node removal method can extract potential network features, the node removal method fails to sufficiently consider the directionality of brain electrical activities. To solve the problems above, this study proposes a feature tensor-based epileptic detection method of directed brain networks. First, a directed functional brain network is constructed by calculating the transfer entropy of EEG signals between different electrodes. Second, the edge removal method is used to imitate the disruptions of brain connectivity, which may be related to the disorder of brain diseases, to obtain a sequence of residual networks. After that, topological features of these residual networks are extracted based on graph theory for constructing a five-way feature tensor. To exploit the inherent interactions among multiple modes of the feature tensor, this study uses the Tucker decomposition method to get a core tensor which is finally reshaped into a vector and input into the support vectors machine (SVM) classifier. Experiment results suggest that the proposed method has better epileptic screening performance for short-term interictal EEG data.

## 1. Introduction

Epilepsy is typically diagnosed by epileptic discharges combined with clinical manifestations of patients (Noachtar and Rémi, 2009). During interictal periods, waveform and rhythm characteristics of epileptic EEG may change as paroxysmal rhythmic slow waves, sharp waves, spikes, or spike-and-wave complexes. Nevertheless, sometimes normal EEG signals account for most of the detection time, which makes it hard to detect epileptic discharges (Pittau et al., 2011; Maganti and Rutecki, 2013). A study stated that the detection rate of epileptic discharge was only 19.2% under the 30-min EEG data when 240 epilepsy patients with conscious resting status were examined (Qin and Dou, 2016). Therefore, long-term interictal EEG data are required for time-domain feature-based methods to improve the detection rate. For example, Krishnan et al. (2014) developed a novel classification method of spike detection to achieve an 87% detection rate of epileptic discharge, but EEG data must be collected for several hours, which is too long for general experiments.

To obtain ideal results in a shorter detection period, the existing approach is to analyze the EEG information interaction between different brain regions, that is, to build brain networks to provide the possibility to distinguish epilepsy patients from healthy controls (Hassan et al., 2017; Najm, 2018; Park et al., 2018). According to the brain network, disruptions of its connectivity are likely to generate brain disorders such as epilepsy, migraine, and schizophrenia (Van den Heuvel and Fornito, 2014; Van Mierlo et al., 2014). Analogically, epileptic discharges are likely to cause abnormal brain network wiring and dynamics. Therefore, exploring the differences in topological features between epileptic and healthy brain networks has become a common method to detect epilepsy, but the detection effect is not always satisfactory (De Lathauwer et al., 2000; Booth, 2005; Subramaniyam and Hyttinen, 2013; Preti et al., 2014; Najm, 2018; Park et al., 2018; Rosch et al., 2018; Li and Cao, 2019).

Moreover, different network structures can lead to different attacking tolerances for edge damage tolerances, so the deliberate attacking methods have been proposed to improve the ability of distinguishing different tolerances network types (Alstott et al., 2009). Typically, most deliberate attacking methods devote themselves to research undirected networks based on node removal (Joyce et al., 2013). The node removal method attempts to analyze the changes of topological features by removing important nodes one after another (Aerts et al., 2016). Different residual networks can be obtained in different removal times, and then the analysis of topological features is naturally generalized from original networks to a sequence of residual networks, and the details of brain networks can be extracted quite adequately (Schlesinger et al., 2017). Nonetheless, the above researches fail to sufficiently take full account of the directionality of brain electrical activities, which makes it hard to reflect the directed information transmission abnormality of epileptic brain networks.

To take directed networks into account more ideally, improved edge removal (ImpER) algorithm, an improved deliberate attacking method for directed networks is proposed in this paper. Furthermore, a feature tensor-based epileptic detection model is designed based on ImpER. After brain network construction, edge removal, Tucker decomposition, and support vector machine (SVM) are employed to feature tensor construction, decomposition, and classification respectively.

When constructing brain networks, transfer entropy (Schreiber, 2000) is exploited to calculate weighted directed edges quantitatively. Transfer entropy is a kind of information theory function reflecting the varying trends of two signals and the dynamic and directional information interaction between two systems (Ma et al., 2018). It has been proved to be very suitable for analyzing time series and has become an increasingly crucial index to measure causality based on predictability and information transfer (Murari et al., 2015). Furthermore, it is the information amount transferred between signals that transfer entropy takes into consideration, which means assumed a specific form of relationship between signals is not necessary (Ma et al., 2018). Hence, it has better applicability in quantitative calculation of brain networks than the Wiener-Granger causality analysis method and mutual information method, especially for non-linear systems (Barnett et al., 2009; Li and Zhang, 2014).

Besides, Lin et al. (2020) also demonstrate that physiologic states can not be fully described by focusing only on individual brain rhythms and on certain pairwise interactions. They discover the dynamic brain networks of interactions among brain rhythms by calculating cross-correlations, but the threshold selection is kind of subjective. Transfer entropy is also a measure of the degree to which two variables are related to each other considering the dynamic process, which emphasizes the amount of information transferred from one variable to another in a period of time.

When it comes to edge removal, rather than analyzing residual networks individually, this study tends to construct feature tensors including topological properties of all residual networks, because tensor analysis technique can fully preserve the multidimensional correlation information.

To improve the generalization ability and reduce the computation costs of classification, the tensor decomposition algorithm should be exploited for extracting principal typical components. In general, typical tensor decomposition methods are CP and Tucker decomposition (Kolda and Bader, 2009). CP decomposition attempts to express a tensor as the sum of a finite number of rank-one tensors. Since there is no finite algorithm for determining the rank of a tensor, it is difficult to determine a suitable number of rank-one tensors (Bro and Kiers, 2003). Therefore, Tucker decomposition is employed in this paper to realize data dimensionality reduction.

Support vector machine (SVM) and its kernel extensions are selected as classifiers in this paper which has been proved to have satisfactory classification accuracy and generalization ability for small-sample and non-linear data (Chauhan et al., 2019). Remarkably, SVM classifiers normally use vectors as input, however, the Tucker decomposition extracts core tensors. Hence, this paper attempts to reshape core tensors into vectors and the performance of these classifiers is evaluated with the method of K-fold cross-validation (Bengio and Grandvalet, 2004).

In this paper, a detailed description of the creation of a feature tensor-based epileptic detection model based on ImpER and its experiments on various groups of subjects is provided. The organization of this paper is as follows: In section 2, we briefly describe the ImpER method. In section 3, we introduce the steps of constructing feature tensors of directed brain networks. In section 4, we conduct experiments on both epilepsy patients and healthy controls and discuss the results. In section 5, we give the conclusions obtained in this paper and the prospects for further research.

## 2. Improved Edge Removal Algorithm

When evaluating the robustness and survivability of an undirected network, the node removal algorithm is adopted to imitate deliberate attack by deleting important nodes from networks in succession (Alstott et al., 2009; Joyce et al., 2013; Aerts et al., 2016; Schlesinger et al., 2017), and the performance of residual networks is analyzed for exploring potential information. By contrast with an undirected network, each node in the directed network plays two different roles simultaneously as a receiver and a sender. When all nodes are regarded as receivers, they can be sorted according to their ability to receive information, and the same is true for the senders. In this section, the node removal algorithm is extended to directed networks in this way, which means improved edge removal (ImpER) algorithm an improved deliberate attacking approach based on edge removal is exploited for directed networks.

### 2.1. Original Node Removal Algorithm for Undirected Network

**Figure 1:**
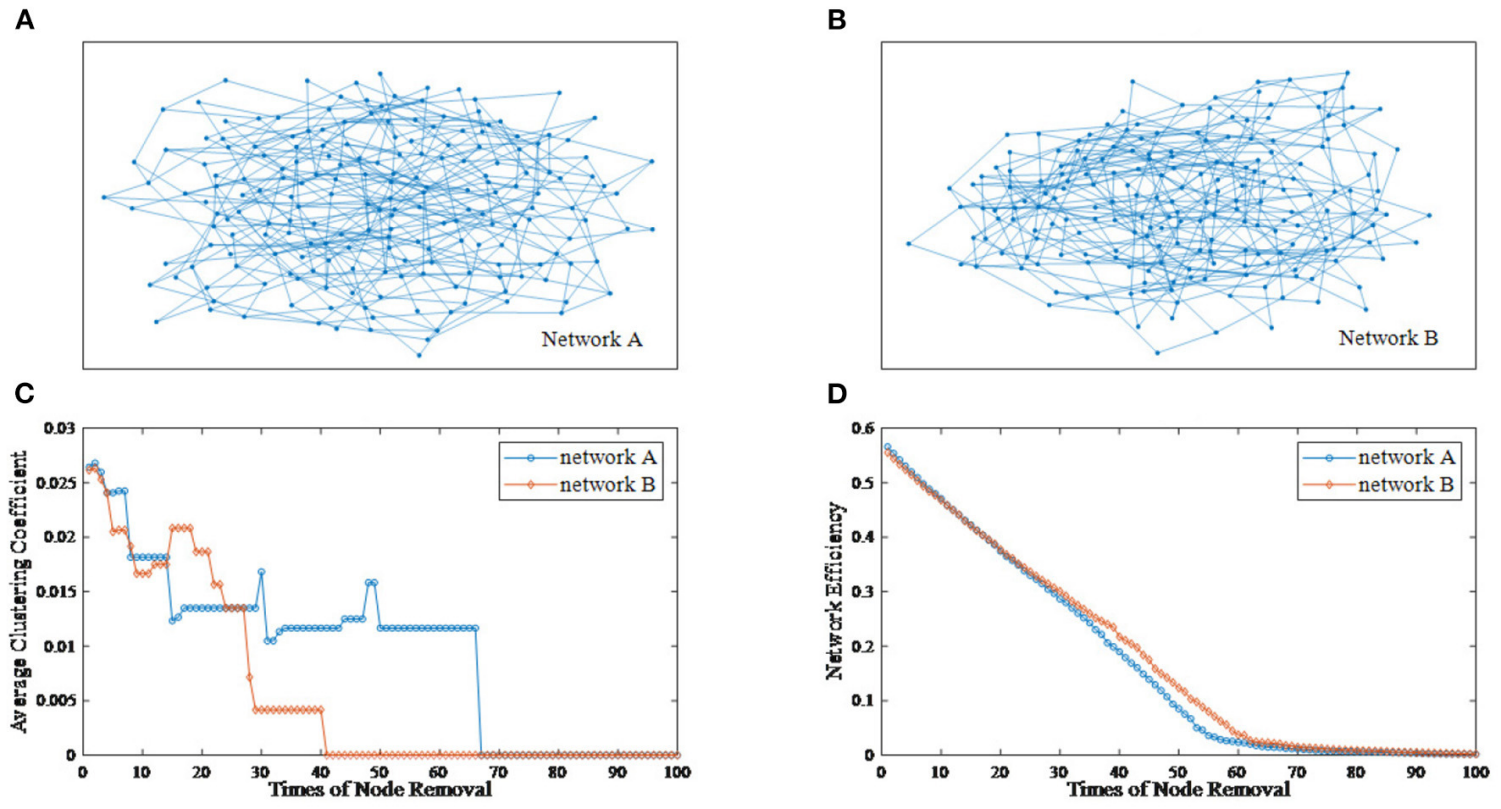
An example of the node removal method. **(A)** Undirected network A. **(B)** Undirected network B. **(C)** The varying curves of the average clustering coefficient with the times of node removal. **(D)** The varying curves of the network efficiency with the times of node removal.

The deliberate attacking theories (Joyce et al., 2013; Aerts et al., 2016; Schlesinger et al., 2017) suggest that, for two undirected networks with similar features, when several important nodes of each network are removed, topological differences between two residual structures may emerge. Figure 1 provides an example of the node removal method, in which the undirected network A and B are randomly generated. Their topological features are shown in Table 1. In this paper, the average clustering coefficient (Strogatz, 2001; Eggemann and Noble, 2011) and the network efficiency (Latora and Marchiori, 2003) are selected to separately reflect functional segregation and integration ability of networks.

**Table 1:** The topological features of Network A and B.

**Topological features**	**Network A**	**Network B**
Average degree	4	4
Average clustering coefficient	0.0264	0.0262
Network efficiency	0.566	0.5551

A network is defined as *G*(*V, E*) whose number of nodes and edges are *n* and *m* respectively. *V* = {*v*_1_, *v*_2_, ⋯ , *v*_*n*_} is the set of nodes, *E* is the set of edges and *e*_*xy*_ is the edge between *v*_*x*_ and *v*_*y*_. *l*_*v*_*i*_*v*_*j*__ is the shortest path length between nodes *v*_*i*_ and *v*_*j*_. For node *v*_*i*_, *C*_*v*_*i*__ is the clustering coefficient, Υ(*v*_*i*_) is the set of neighbor nodes, and *k*_*v*_*i*__ is the degree of node *v*_*i*_ respectively. Above all, the average degree *K*, the network efficiency *N*, and the average clustering coefficient *C* can be defined as:

(1) $$\begin{array}{l} K=\frac{1}{n}\underset{v_{i}\in V}{\sum }k_{v_{i}}, \end{array}$$

(2) $$\begin{array}{l} N=\frac{1}{n\left(n-1\right)}\underset{v_{i}\neq v_{j}\in V}{\sum }\frac{1}{l_{v_{i}v_{j}}}, \end{array}$$

(3) $$\begin{array}{l} C=\frac{1}{n}\underset{v_{i}\in V}{\sum }C_{v_{i}}\\ \;=\{\begin{array}{ll} \frac{1}{n}\sum_{v_{i}\in V}\frac{2\left|\left\{e_{xy}|v_{x}\in ϒ(v_{i}),v_{y}\in ϒ(v_{i}),e_{xy}\in E\right\}\right|}{k_{v_{i}}(k_{v_{i}}-1)}, & \left(k_{v_{i}}\geq 2\right)\\ 0, & \left(k_{v_{i}}<2\right) \end{array}. \end{array}$$

From Figure 1, we can see that the differences between network A and B is subtle. Now, we regard the degree as the node importance evaluation and attempt to remove one node with the largest degree at a time. Figures 1C,D show the varying curves of topological features with the times of node removal. Obviously, there are significant differences in the variation trend of the average clustering coefficient between network A and B. Furthermore, after removing 35 nodes, the network efficiency of network B is obviously higher than that of network A. This example shows that the node removal method is effective to explore potential information of undirected networks.

### 2.2. Improved Edge Removal Algorithm for Directed Network

For directed networks, there are two types of edges connected to a node: receiving edges and sending edges. At this point, the node removal method, which removes all edges connected to a node, neglects the directionality of edges. Therefore, this study attempts to design an improved deliberate attacking method for directed networks based on edge removal. Here, nodes of a directed network are considered to play two different roles simultaneously as receivers and senders.

***Sub-approach* I. Receiving-Edge Removal (Re-ER)**. When all nodes are regarded as receivers, prohibit the node with the strongest receiving capability from receiving information, which means all of the edges pointing to this node are deleted to obtain the First Residual Network (1*stRN*). Next, repeat this process in the 1*stRN* to obtain the Second Residual Network (2*ndRN*). Finally, repeat this step continuously until all nodes are deprived of the receiving capability, which means all edges in the network have been deleted. Hence, the Last Residual Network (*nthRN*) is formed with isolated nodes. At this point, a sequence of residual networks can be obtained. Above all, this removal sub-approach is named receiving-edge removal.

***Sub-approach* II. Sending-Edge Removal (Se-ER)**. When all nodes are regarded as senders, all processes are similar to Re-ER, only substituting sending for receiving. Also, this removal sub-approach is called sending-edge removal.

**Figure 2:**
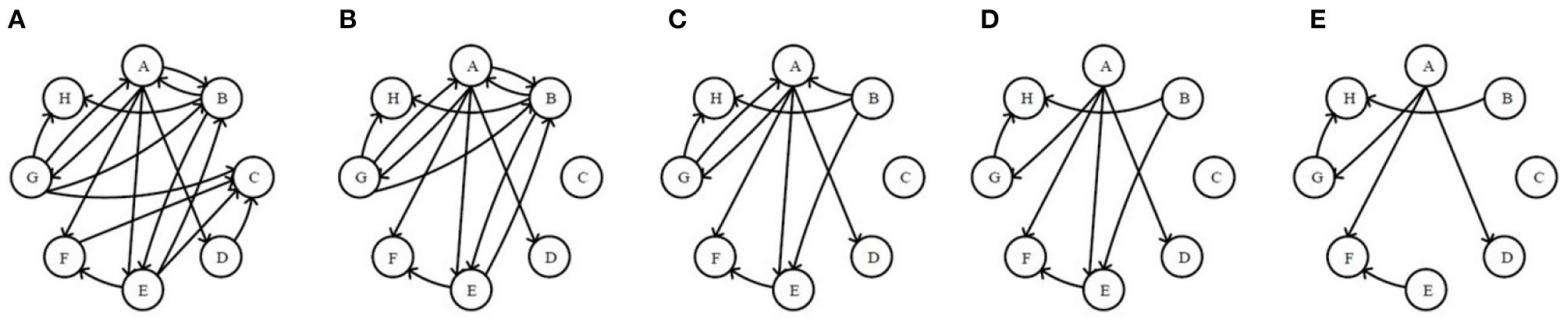
The original network and the first four residual networks based on receiving-edge removal. **(A)** The original network. **(B)** The 1*stRN*. **(C)** The 2*ndRN*. **(D)** The 3*rdRN*. **(E)** The 4*thRN*.

To measure the directionality of network nodes well, in-degree and out-degree are employed as relevant indices in this paper. A directed network with 8 nodes in Figure 2A are randomly generated, to which both Re-ER and Se-ER method in the process will be applied in the next three steps.

***Step* I. Sorting**. Sort receiving and sending capability of nodes based on their in-degree and out-degree from high to low, as shown in Table 2.

**Table 2:** The order of nodes' receiving and sending capability.

Receiving capability	C	B	A	E	F	H	D	G
(In-degree)	4	3	2	2	2	2	1	1
Sending capability	A	G	B	E	D	F	C	H
(Out-degree)	5	4	3	3	1	1	0	0

***Step* II. Application of Re-ER**. Now, all nodes are considered as receivers. First, delete all of the edges pointing to node C to obtain the 1*stRN*. Next, delete all of the edges pointing to node B to obtain the 2*ndRN*, and node *A, E, F, H, D, G* will also be operated sequentially in this way. In the process, the original network and the first four residual networks are exhibited in Figure 2.

**Figure 3:**
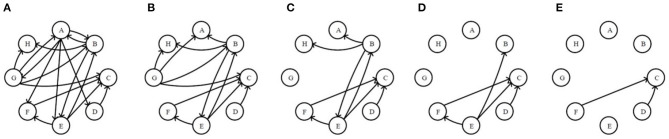
The original network and the first four residual networks based on sending-edge removal. **(A)** The original network. **(B)** The 1*stRN*. **(C)** The 2*ndRN*. **(D)** The 3*rdRN*. **(E)** The 4*thRN*.

***Step* III. Application of Se-ER**. The operations are completed in a similar way like ***Step*** **II** according to sending capability, as shown in Figure 3.

In Figures 2, 3, it is obvious that the network is becoming sparser with the increasing of the edge removal times. Moreover, the residual network structures with Re-ER and Se-ER are different, so the topological features of two residual network sequences can be analyzed separately.

Similar to the node removal method, the varying curve of topological features with the times of edge removal can be obtained based on the two sub-methods respectively. The network efficiency *N* and the average clustering coefficient *C* are also exploited to evaluate local and global topological features. Remarkably, for directed networks, the average clustering coefficient *C* should be rewritten as follows.

(4) $$C=\frac{1}{n}\sum_{v_{i}\in V}C_{v_{i}}=\{\begin{array}{ll} \frac{1}{n}\sum_{v_{i}\in V}\frac{\left|\left\{e_{xy}|v_{x}\in ϒ(v_{i}),v_{y}\in ϒ(v_{i}),e_{xy}\in E\right\}\right|}{k_{v_{i}}(k_{v_{i}}-1)}, & \left(k_{v_{i}}\geq 2\right)\\ 0, & \left(k_{v_{i}}<2\right) \end{array}.$$

The network efficiency *N* is also supposed to embody the directionality, *l*_*v*_*i*_*v*_*j*__ is the shortest path length from nodes *v*_*i*_ to *v*_*j*_, but not between themselves.

**Figure 4:**
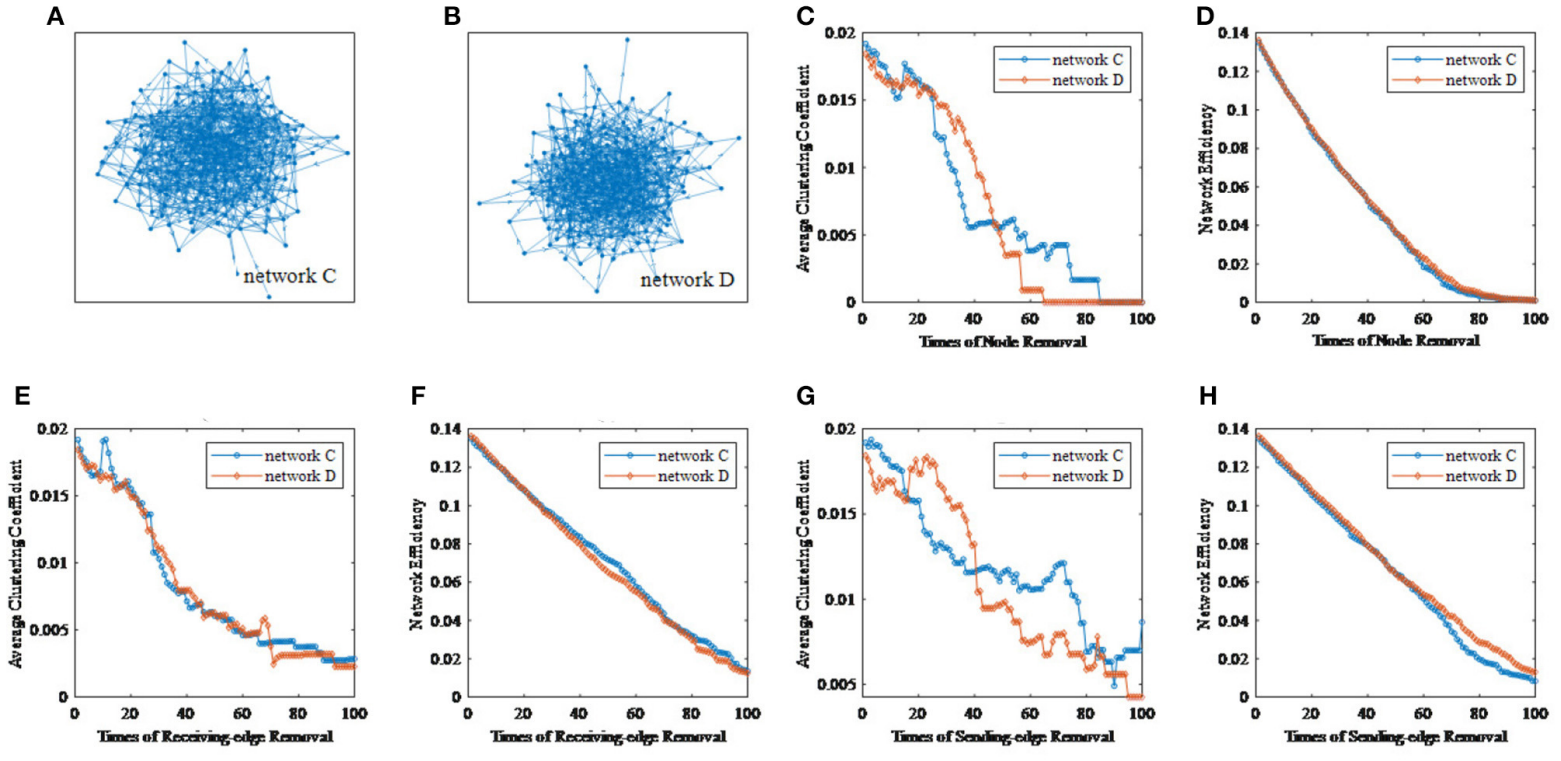
The comparisons of the node removal and the edge removal method in exploring potential information of directed networks. **(A)** Directed network C. **(B)** Directed network D. **(C)** The varying curves of the average clustering coefficient with the times of node removal. **(D)** The varying curves of the network efficiency with the times of node removal. **(E)** The varying curves of the average clustering coefficient with the times of receiving-edge removal. **(F)** The varying curved of the network efficiency with the times of receiving-edge removal. **(G)** The varying curves of the average clustering coefficient with the times of sending-edge removal. **(H)** The varying curves of the network efficiency with the times of sending-edge removal.

To illustrate the effectiveness of the edge removal method, we compare it with the traditional node removal method. Figure 4 randomly generates two directed networks with 200 nodes as network C and network D, whose topological features are shown in Table 3. Figures 4C,D are the results of the node removal method, and Figures 4E–H are the results of the edge removal method.

**Table 3:** The Topological features of Network C and D.

**Topological features**	**Network C**	**Network D**
Average degree	7.87	7.61
Average in-degree	3.935	3.805
Average out-degree	3.935	3.805
Average clustering coefficient	0.0192	0.0184
Network efficiency	0.135	0.1361

For the node removal method, the network efficiency curves of network C and D are extremely similar. By contrast, based on the Re-ER, after removing 40 nodes, the network efficiency of network C is higher than that of network D. Meanwhile, based on the Se-ER, after removing 60 nodes, the network efficiency of network D is obviously higher than that of network C. Additionally, whether based on the Re-ER or the Se-ER, there are significant differences in the average clustering coefficient between network C and D. Hence, compared with the node removal method, the edge removal method can more fully explore the intrinsic information of directed networks.

## 3. Feature Tensor-based Epilepsy Detection Method

Due to the differences between effects of the Re-ER and Se-ER on directed networks, to integrate the results of these two sub-approaches, a feature tensor-based epilepsy detection model of directed brain networks is designed in this paper, of which the six steps are as follows.

***Step* I**. EEG signals from different electrodes are supposed to be collected simultaneously.

***Step* II**. Transfer entropy is employed to calculate weighted directed edges quantitatively.

***Step* III**. The weighted network needs to be binarized into a certain number of unweighted sparse networks based on multiple thresholds.

***Step* IV**. We can use these unweighted networks to construct a five-way feature tensor with the edge removal approach.

***Step* V**. We attempt to extract a core component from the feature tensor by the Tucker decomposition method for epilepsy detection. The whole model is described in detail below.

***Step* VI**. Epileptic brain networks can be distinguished from healthy ones by using Support Vector Machine (SVM) classifiers.

**Figure 5:**
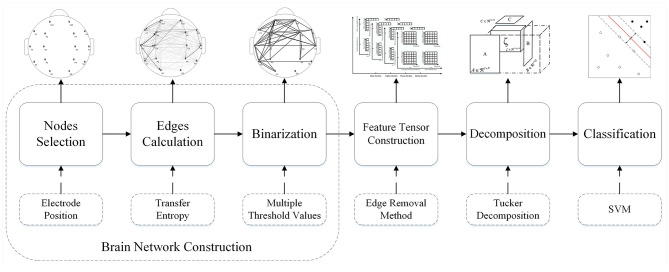
Flowchart of the feature tensor-based epileptic detection model of brain networks based on edge removal.

In this six-step process, the first three steps consist of the Brain Network Construction, which will be stated in section 3.1 detailedly. Comprehensively, an integral flowchart is displayed in Figure 5.

### 3.1. Brain Network Construction

First, we should simultaneously collect EEG signals from different electrodes. Since EEG rhythms are related to specific cerebral functions of different brain regions (Gastaut et al., 1985; Duque-Munoz et al., 2013; Pyrzowski et al., 2015), original EEG signals are decomposed into different rhythm components as β (13–30 Hz), α (8–13 Hz), θ (4–8 Hz), and δ (1–4 Hz) rhythm.

Next, we attempt to respectively build weighted directed brain networks in each EEG rhythm. The widely used EEG directed connectivity measurement methods are Wiener-Granger causality (Korzeniewska et al., 2003; Blinowska et al., 2004; Bressler and Seth, 2011) and transfer entropy (Schreiber, 2000). To obtain accurate results, the calculation of Wiener-Granger causality must satisfy three prerequisites as: **I**. The interaction between the two signals should be approximately linear. **II**. The noise of observations should be relatively weak. **III**. The crosstalk between the two signals should be weak.

Nevertheless, interactions between EEG signals are always non-linear, and if EEG electrodes are placed on the scalp, due to the volume conduction of human heads (volume conductors), a single-channel EEG signal always contains causally-related brain signals from different sources. Coupled with noise and artifacts, it is difficult for Wiener-Granger causality to estimate information flow between brain regions.

By contrast with Wiener-Granger causality, transfer entropy is not absolutely bound by the above three prerequisites and suffer from problems of non-linearity, data noise and cross talk to a lesser extent, which means transfer entropy is robust to volume conduction (Astolfi et al., 2007; Gourévitch and Eggermont, 2007; Supp et al., 2007; Sabesan et al., 2009; Besserve et al., 2010; Lee et al., 2012). Therefore, transfer entropy is employed in this paper to reveal non-linear interactions. Transfer entropy is proposed to evaluate conditional transition probabilities between two signals evolving. Suppose two simultaneously measured EEG signals, which can be approximated by a stationary Markov process of finite order *d*, as *X* = *x*_*t*_ and *Y* = *y*_*t*_. At this point, reconstruct the state space of *X* by a delay τ embedded vector of dimension *d* as $X_{t}^{d}=\left(x_{t},x_{t-\tau },x_{t-2\tau },\cdots \;,x_{t-\left(d-1\right)\tau }\right)$. Since *p*(·) is regarded as the probability, the transition probabilities of *X* can be written as $p\left(x_{t+1}|X_{t}^{d}\right)$. Accordingly, when the prediction time is *u*, the entropy rate *H*(·) can be computed as:

(5) $$\begin{array}{l} H\left(x_{t+u}|X_{t}^{d}\right)=-\underset{X_{t+u},X_{t}^{d}}{\sum }p\left(x_{t+u},X_{t}^{d}\right)\log p\left(x_{t+u}|X_{t}^{d}\right), \end{array}$$

(6) $$\begin{array}{l} p\left(x_{t+u}|X_{t}^{d}\right)=p\left(x_{t+u},X_{t}^{d}\right)/p\left(X_{t}^{d}\right), \end{array}$$

which represents the average number of bits required to obtain an additional state if all previous states are known. Now, a directed measure of information transfer from Y to X can be computed based on Kullback divergence or mutual information (Van Erven and Harremos, 2014; Gabrié et al., 2018). At this point, the transfer entropy *T*—amount of information transferred from *Y* to *X*, can be expressed as:

(7) $$\begin{array}{l} T\left(Y\to X\right)=\sum p\left(x_{t+u},X_{t}^{d},Y_{t}^{m}\right)\log\frac{p\left(x_{t+u}|X_{t}^{d},Y_{t}^{m}\right)}{p\left(x_{t+u}|X_{t}^{d}\right)}, \end{array}$$

(8) $$\begin{array}{l} Y_{t}^{m}=\left(y_{t},y_{t-\tau },\cdots \;,y_{t-\left(m-1\right)\tau }\right), \end{array}$$

where $Y_{t}^{m}$ indicates that *X* depends on *m* states of *Y*. Based on the differential entropy, the transfer entropy can be rewritten as:

(9) $$\begin{array}{l} T\left(Y\to X\right)=H\left(X_{t}^{d},Y_{t}^{m}\right)-H\left(x_{t+u},X_{t}^{d},Y_{t}^{m}\right)+H\left(x_{t+u},X_{t}^{d}\right)-H\left(X_{t}^{d}\right). \end{array}$$

Remarkably, transfer entropy can indicate the direction of information transmission, ordinarily *T*(*Y* → *X*) ≠ *T*(*X* → *Y*). Also, when *X* and *Y* are independent, *T*(*Y* → *X*) ≠ *T*(*X* → *Y*) = 0.

Since the constructed brain networks are directed, weighted, and fully connected, to adapt to the edge removal method, it is necessary to set thresholds to obtain unweighted networks. Nonetheless, choosing one threshold may ignore useful information under other thresholds, this paper attempts to select multiple thresholds rather than a single one.

### 3.2. Feature Tensor Construction Based on ImpER

**Figure 6:**
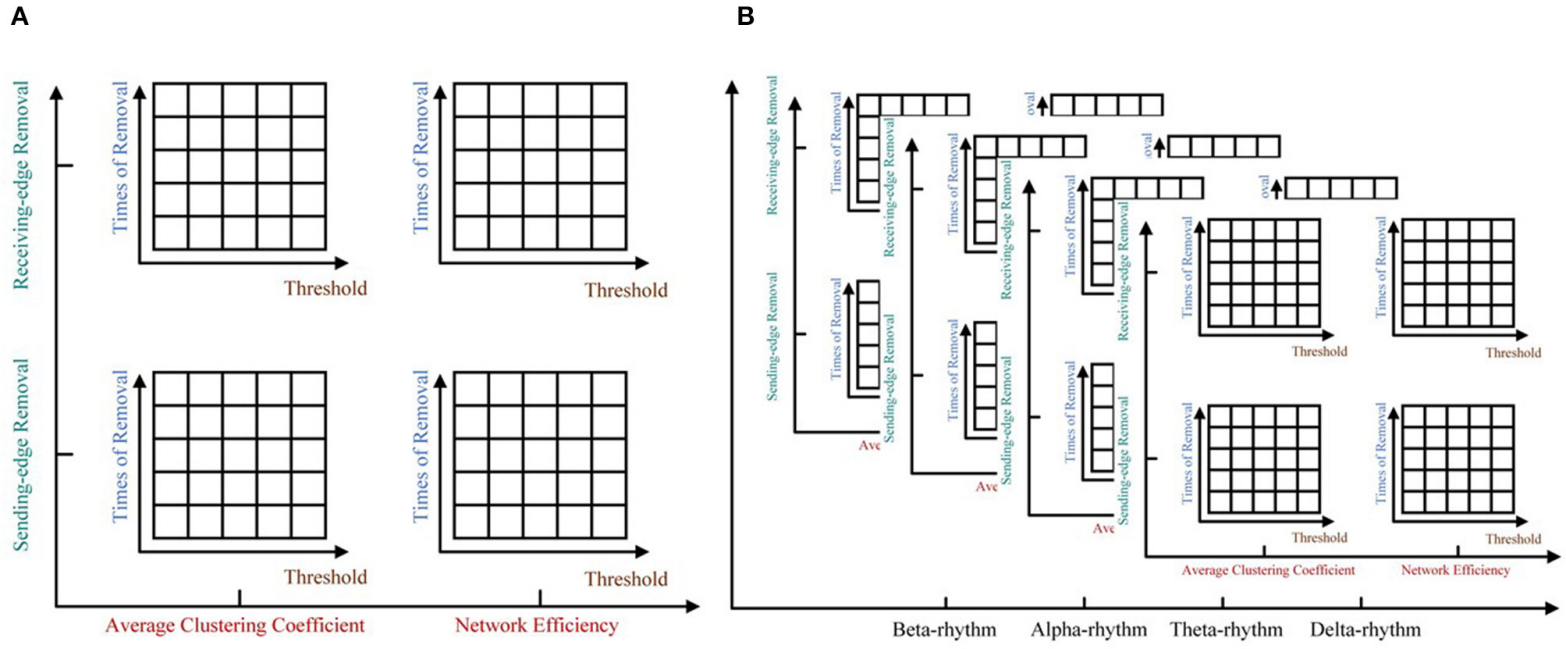
The five-way feature tensor. **(A)** The average clustering coefficient and the network efficiency can be calculated respectively based on different threshold values, different times of edge removal and different edge removal sub-methods. **(B)** The five-way feature tensor of brain networks.

For each rhythm, we can get a certain number of unweighted sparse networks, each of whom can be used to get a large number of topological features based on the ImpER method. To avoid damaging the relationship among features, we attempt to use the tensor method to organize them, as shown in Figure 6.

To be more specific, five-way feature tensors is obtained as $Q\in R^{\epsilon_{1}\times \epsilon_{2}\times \epsilon_{3}\times \epsilon_{4}\times \epsilon_{5}}$, and ϵ_1_ to ϵ_5_, respectively indicates threshold, removal time, edge removal sub-approach, topological feature, and EEG rhythm. If the number of thresholds and network nodes are set as *h* and *n*, the size of feature tensors should be *h* × *n* × 2 × 2 × 4 (two edge removal sub-methods as the receiving and sending-edge removal sub-method, two topological features as the average clustering coefficient and the network efficiency, and four EEG rhythms as the β, α, θ, and δ rhythm). For example, the < 1, 1, 1, 1, 1 >^*th*^ element of *Q* represents the average clustering coefficient of a network which have been attacked for one time with the Re-ER sub-approach, and the original network is constructed by beta EEG rhythm and binarized under the first threshold.

### 3.3. Tensor Decomposition and Classification

**Figure 7:**
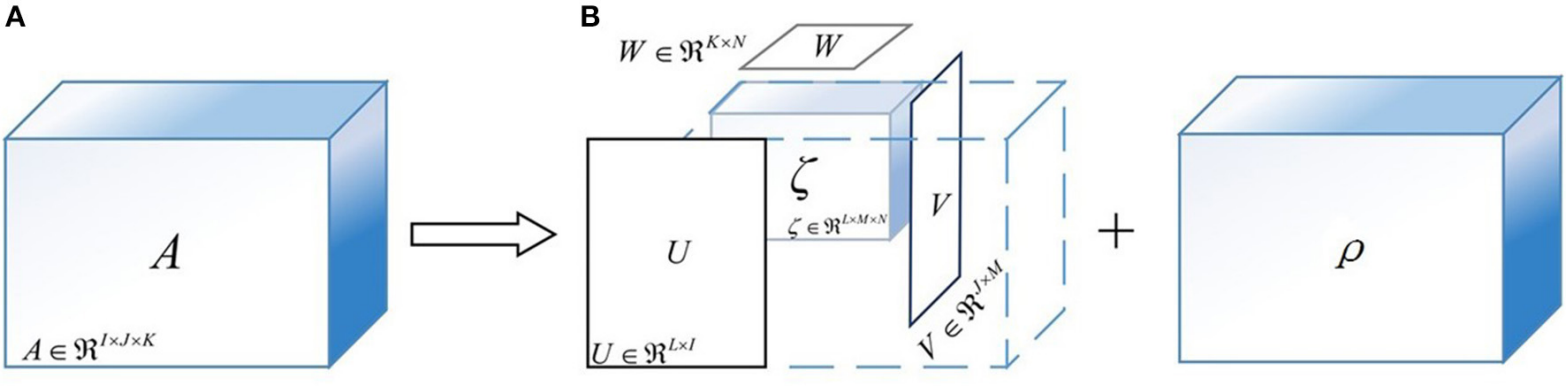
The sketch maps of Tucker decomposition for a three-way tensor. **(A)** The original feature tensor A. **(B)** The result of Tucker decomposition algorithm. **(Note)** *U*, *V*, and *W* are the factor matrices; ζ is the core tensor; ρ is the approximation error.

Tucker decomposition is the higher-order generalization of the singular value decomposition, which attempts to decompose a tensor into several factor matrices and a lower-dimensional core tensor (Kolda and Bader, 2009). To graphically demonstrate the principle of Tucker decomposition, Figure 7 is illustrated with a three-way tensor as an example. As shown in Figure 7, the three-way tensor *A* ∈ *R*^*I*×*J*×*K*^ can be decomposed as:

(10) $$\begin{array}{l} A=\zeta \times_{1}U\times_{2}V\times_{3}W+\rho , \end{array}$$

where ζ ∈ *R*^*L*×*M*×*N*^ is the core tensor, being generally smaller than *A*. Moreover, *U* ∈ *R*^*L*×*I*^, *V* ∈ *R*^*J*×*M*^, and *W* ∈ *R*^*K*×*N*^ are the factor matrices; ρ ∈ *R*^*I*×*J*×*K*^ is the error term and ×_*i*_ is the product of a tensor and a matrix along mode-*i*.

Since the purpose of applying Tucker decomposition in this paper is to extract core components for the epileptic detection, but not to restore the original tensors, it is unnecessary to find the exact Tucker decomposition, and excessively fine features are not conducive to the generalization ability of classification models. Furthermore, to guarantee the classification algorithms, the core tensors are supposed to be set to the same size. Therefore, this paper attempts to extract core tensors Λ = λ_1_×λ_2_×λ_3_×λ_4_×λ_5_, whose size is smaller than the original feature tensors *Q*. For data dimensionality reduction, λ_*i*_(*i* = 1, 2, ⋯ , 5) should be greater than 1 but less than 10. Since the size of *Q* is *u* × *n* × 2 × 2 × 4, we can get that λ_3_ = 2, λ_4_ = 2, and λ_5_ can be taken as 2, 3, or 4. In addition, if *u* ≥ 10, λ_1_ can be taken as 2, 3, ⋯ , 9; otherwise λ_1_ = 2, 3, ⋯ , *h*, and the possible value of λ_2_ is similar to that of λ_1_. Therefore, there are 3×*min*(*u*−1, 8)×*min*(*n*−1, 8) selections of core tensors size. To look for the global optimal solution, this paper adopts the complete trial scheme, namely, considering all possible combinations.

To make core tensors Λ represent *Q* as fully as possible, this paper uses the fraction ψ explained by model (Bader and Kolda, 2006) as:

(11) $$\begin{array}{l} \psi =1-\frac{\sqrt{\Gamma {\left(Q\right)}^{2}-\Gamma {\left(\Lambda \right)}^{2}}}{\Gamma \left(Q\right)}, \end{array}$$

where Γ· is the norm of tensors. Hence, higher-order orthogonal iteration is used to compute the best n-rank approximation of *Q* and the number of algorithm iterations should be based on ψ, but lower than an upper limit.

Since the number of both positive and negative samples used in this paper is small (50 epileptic and 50 normal persons described in section 4, SVM and its kernel extensions are selected as classifiers in this paper which has been proved to have satisfactory classification accuracy and generalization ability for small-sample and non-linear data (Chauhan et al., 2019). Remarkably, SVM classifiers normally use vectors as input, however, the Tucker decomposition extracts core tensors. Hence, this paper attempts to reshape core tensors into vectors and the performance of these classifiers is evaluated with the method of K-fold cross-validation (Bengio and Grandvalet, 2004).

## 4. Experimental Verification and Results

### 4.1. Data Recording and Pre-processing

The subjects of this study were 50 epileptic patients (25 females, 25 males; the mean age: 29.59 ± 4.34 years) and 50 healthy controls (25 males, 25 females; the mean age: 26.86 ± 3.69 years). EEG data of those subjects were collected by NeuroTop NT9200 (SYMTOP instrument Co. Ltd. China) at the Neurology Department of the General Hospital of Eastern Theater Command (approval number [2016NZGKJ-021]). All the patients were diagnosed with epilepsy by at least 2 qualified neurologists after reviewing their medical records systematically. The 30 controls were selected from volunteers who were matched to patients based on age and gender. All participants were right-handed with no history of smoking, diabetes, head trauma, alcohol or drug abuse, and substance dependence, and they were fully informed about this experiment, signed the written consent form before this experiment. When EEG data were acquired from 16 scalp loci (*Fp*1, *Fp*2, *F*3, *F*4, *C*3, *C*4, *P*3, *P*4, *O*1, *O*2, *F*7, *F*8, *T*3, *T*4, *T*5, *T*6) with Ag/AgCl electrodes which were placed per the international standard 10–20 system (thus the total number of nodes *n* = 16). Every participant with eyes closed was sitting in a dimly lit, electromagnetic shield, and noiseless laboratory. The sampling frequency was set to 512 Hz and recording time was longer than 2 min. Furthermore, power interference of 50 Hz was eliminated. After eliminating the apparent problems like the obvious signal loss, the EEG data of more than 2 min are divided according to a period of every 20 s.

The pretreatment steps in the analysis of EEG signals included reducing ambient noise and removing artifacts including EMG, EOG, and ECG based on the ICA algorithm. Then the sampling frequency was reduced to 256 Hz for computation reduction. The preprocessed EEG signals were decomposed into four rhythms as β, α, θ, and δ rhythm by *db*4 wavelet packet.

### 4.2. Results and Discussion

**Figure 8:**
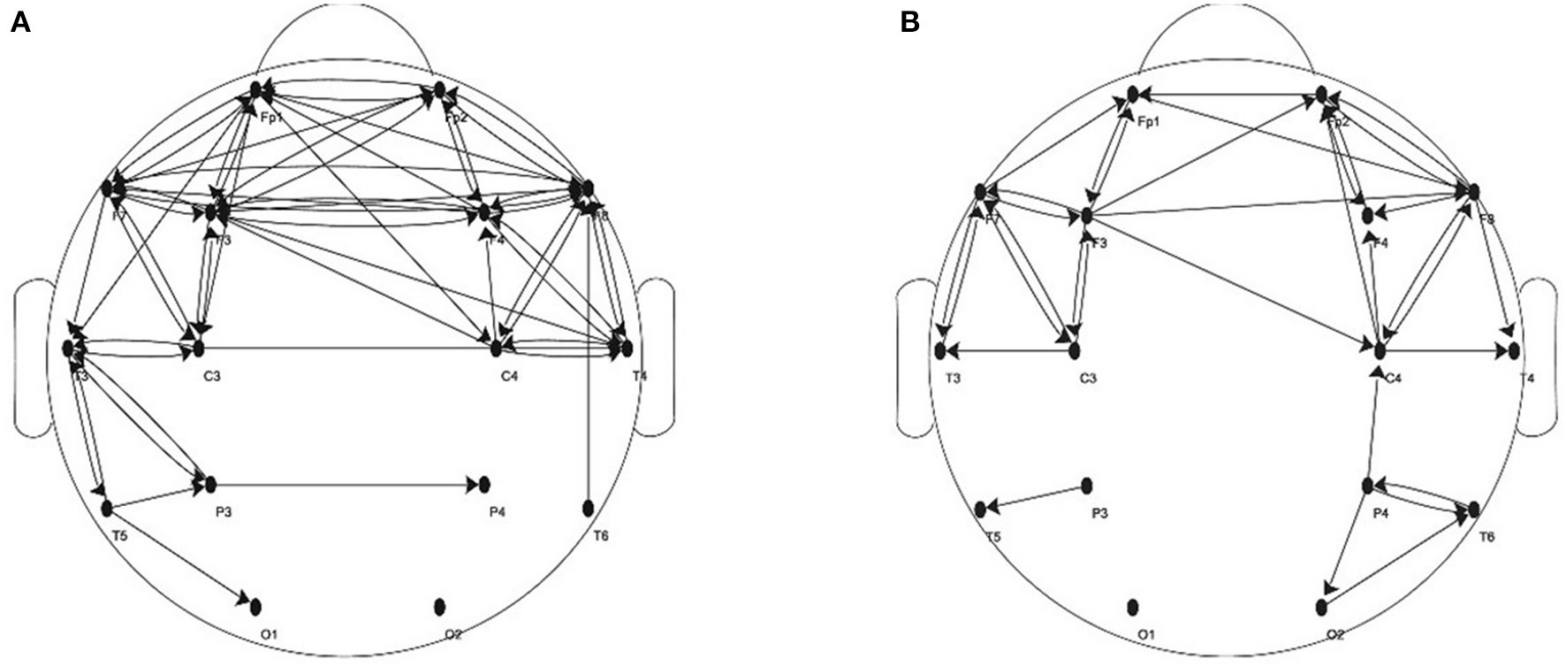
When the threshold is 0.003. **(A)** Directed binarized network of healthy sample in α rhythm. **(B)** Directed binarized network of epileptic sample in α rhythm.

Based on transfer entropy, weighted brain networks of different EEG rhythms can be constructed. Before training the SVM classifier, this study attempts to take the weighted networks of 50 epileptic patients as a sample, and then to apply the right-tailed T-test to the edge weight sample of each pair of nodes. If the edge weight sample of one pair of nodes is significantly greater than the threshold (Significance level *P* < 0.01), these two nodes are considered to connect. In this way, we can construct an epileptic sample brain network under a certain threshold. As mentioned in section 3.1, this study sets a fixed step to get multiple thresholds. The fixed step is 5–10 which makes the threshold increase from 0 to 0.006 (i.e., 600 thresholds). Healthy sample brain networks can be obtained in the same way. Figure 8 schematically shows the healthy and the epileptic sample brain network in alpha rhythm when the threshold is 0.003. Then the methods of receiving-edge and sending-edge removal can be used to obtain a series of residual networks for extracting topological features.

**Figure 9:**
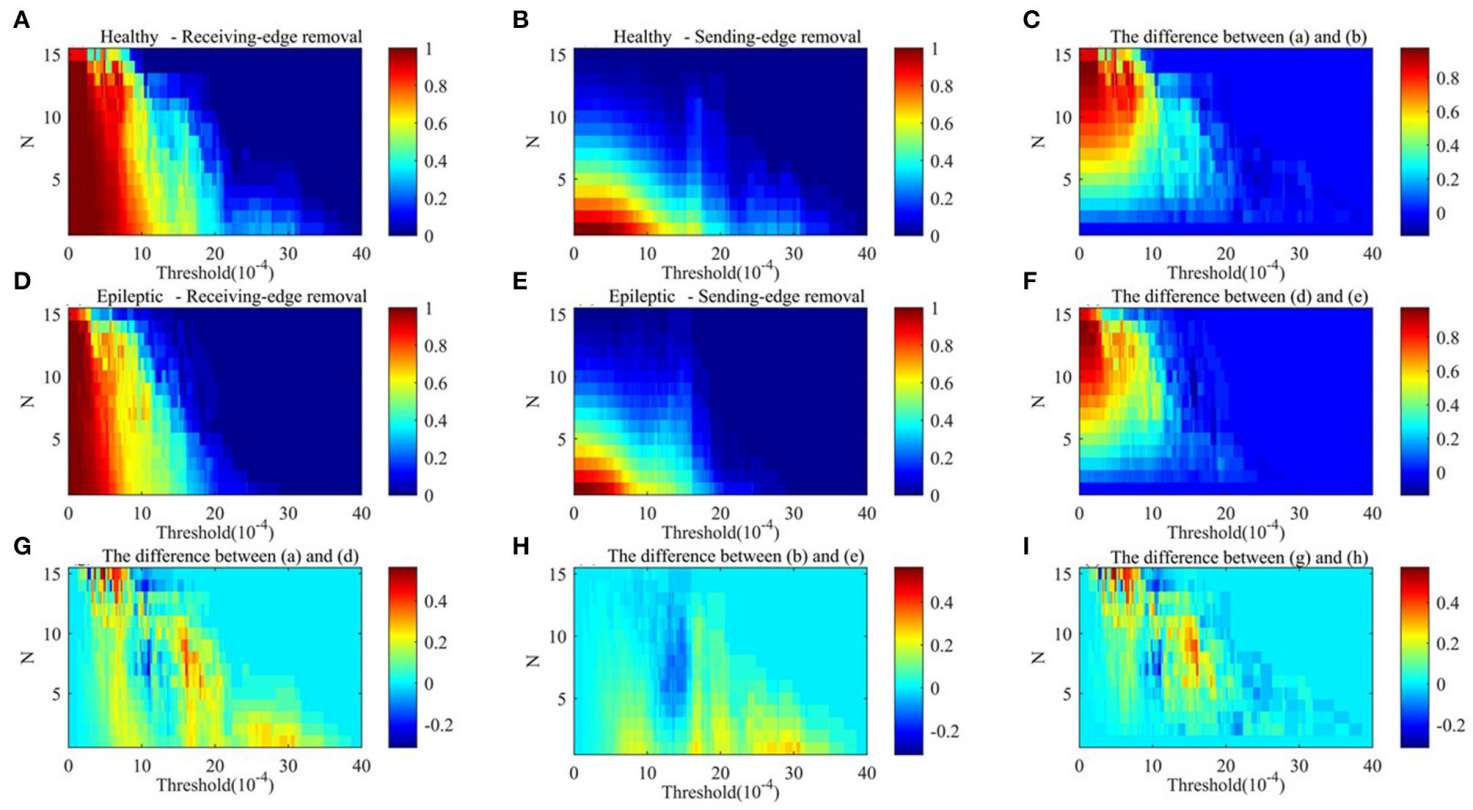
The average clustering coefficient of residual networks in alpha rhythm. **(A)** Healthy sample brain networks based on receiving-edge removal. **(B)** Healthy sample brain networks based on sending-edge removal. **(C)** The difference between **(A)** and **(B)**. **(D)** Epileptic sample brain networks based on receiving-edge removal. **(E)** Epileptic sample brain networks based on sending-edge removal. **(F)** The difference between **(D)** and **(E)**. **(G)** The difference between **(A)** and **(D)**. **(H)** The difference between **(B)** and **(E)**. **(I)** The difference between **(C)** and **(F)**.

**Figure 10:**
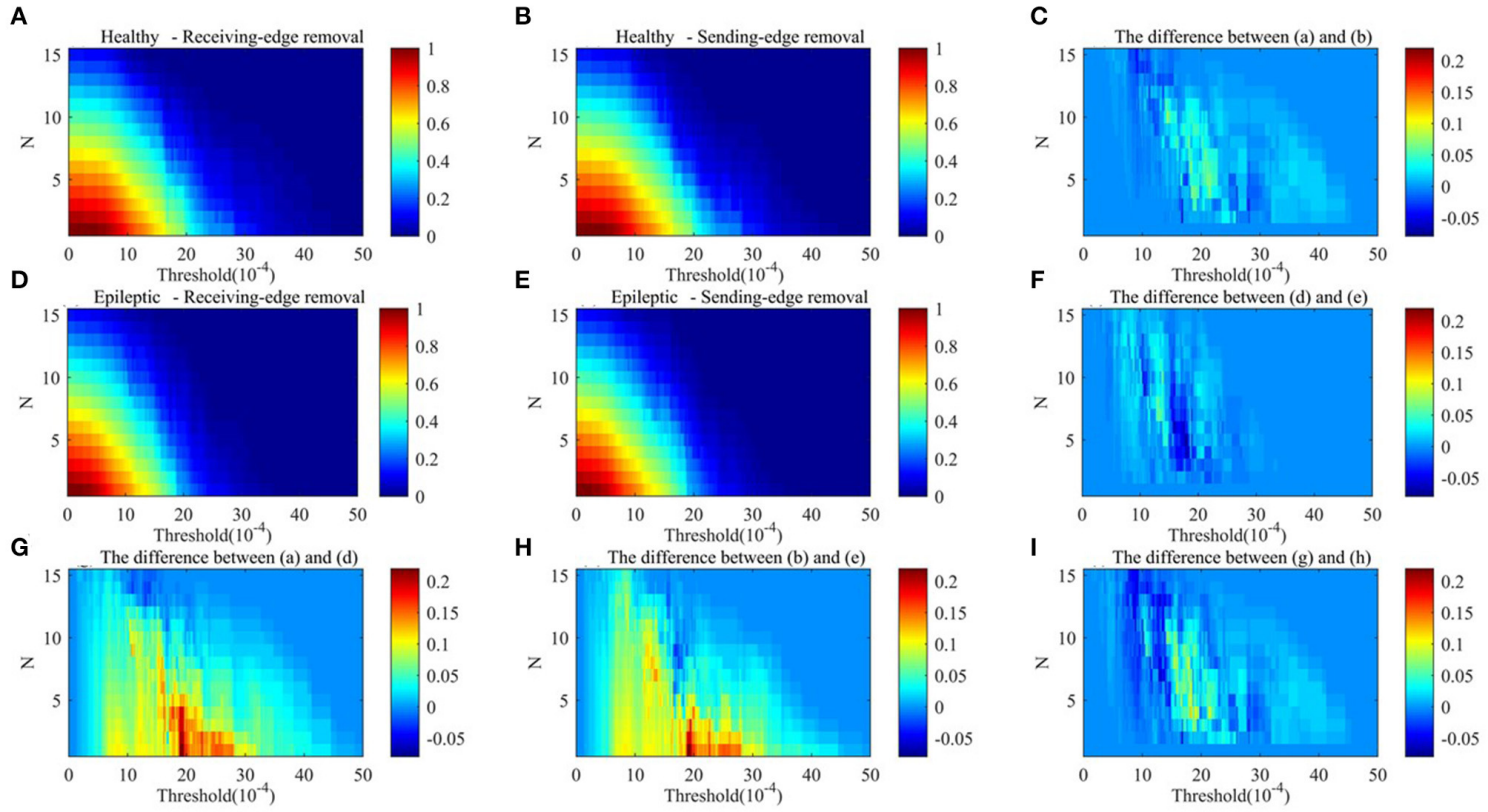
The network efficiency values of residual networks in alpha rhythm. **(A)** Healthy sample brain networks based on receiving-edge removal. **(B)** Healthy sample brain networks based on sending-edge removal. **(C)** The difference between **(A)** and **(B)**. **(D)** Epileptic sample brain networks based on receiving-edge removal. **(E)** Epileptic sample brain networks based on sending-edge removal. **(F)** The difference between **(D)** and **(E)**. **(G)** The difference between **(A)** and **(D)**. **(H)** The difference between **(B)** and **(E)**. **(I)** The difference between **(C)** and **(F)**.

Figures 9, 10 respectively show the average clustering coefficient and the network efficiency of residual networks. These residual networks are generated by epileptic and healthy sample brain networks based on different thresholds, edge removal sub-approaches, and times of edge removal in alpha rhythm. The horizontal axis in every sub-graph represents the thresholds and the vertical axis represents the times of edge removal.

Figures 9, 10 indicate, along with the increase of the thresholds and the edge removal times, topological parameters of epileptic and healthy sample brain networks have the same changing trend. However, for both Re-ER and Se-ER sub-approach, when the thresholds are set between 0.001 and 0.0015, healthy sample networks have a lower average clustering coefficient than epileptic ones. Moreover, when the thresholds are set between 0.002 and 0.003 and the edge removal times are between 0 and 5, network efficiencies of healthy sample networks are larger than those of epileptic ones.

Now we attempt to construct five-way feature tensors of every 50 epileptic patients and 50 healthy controls with the edge removal method, and then to decompose these feature tensors by Tucker decomposition algorithm. The five-way feature tensor is *X* ∈ *R*^600 ×16 ×2 ×2 ×4^, because there are 600 different thresholds, 16 times of edge removal (16 network nodes) and 4 EEG rhythms, and the core tensors are set to ζ ∈ *R*^8 ×6 ×2 ×2 ×4^. All the core tensors can be reshaped into 100 labeled (*epileptic* and *healthy*), 768-dimensional feature vectors for training the SVM classifier.

The performance of classifiers is evaluated in terms of *sensitivity*, *specificity*, and *accuracy* (Stehman, 1997), which are all statistical measures of the performance of a binary classification test. The *sensitivity* measures the proportion of epileptic patients who are correctly identified, that is defined as:

(12) $$\begin{array}{l} sensitivity=\frac{TP}{TP+FN}, \end{array}$$

where *TP* is *True* *Positive* the number of epileptic patients correctly identified as epilepsy and *FN* is *False* *Negative* the number of epileptic patients incorrectly identified as healthy. Similarly, the *specificity* measures the proportion of healthy controls that are correctly identified. It is defined as:

(13) $$\begin{array}{l} specificity=\frac{TN}{TN+FP}, \end{array}$$

where *TN* is *True* *Negative* the number of healthy controls correctly identified as healthy and *FP* is *False* *Positive* the number of healthy controls incorrectly identified as epilepsy. The *accuracy* is a measure of statistical bias, and it is defined as:

(14) $$\begin{array}{l} accuracy=\frac{TP+TN}{TP+FP+FN+TN}. \end{array}$$

The performance of all classifiers is evaluated based on the mean results of twenty 10-fold cross-validations. It shows that the classification results when λ_5_ = 3 are generally lower than those when λ_4_ = 2*or*4. Besides, the results are also unsatisfactory when the values of λ_1_ and λ_2_ are too large or small. Through comparative analysis, when λ_1_ = 6 and λ_2_ = 4, namely the size of core tensors is 6×4×2×2×3, SVM classifiers perform best and the results are shown in Table 4. we can see that the performance of the linear kernel is mediocre, while the coarse Gaussian kernel performs well insensitivity, but do less well in specificity, and the opposite performance can be found in coarse Gaussian kernel. In general, the classification performance of medium Gaussian kernel is the best, and the *sensitivity*, *specificity*, and *accuracy* are all more than 86%.

**Table 4:** The SVM performance compare with different kernel for the proposed method.

	**Sensitivity %**	**Specificity %**	**Accuracy %**
Linear	73.30	70.00	71.70
Fine Gaussian	60.00	93.30	76.70
Medium Gaussian	86.60	90.00	88.30
Coarse Gaussian	96.70	43.30	70.00

Table 5 compares the proposed method with other studies from three perspectives as *accuracy*, *signallength*, *thenumberofchannels*, and the *typeofsignal*. The result shows that the method proposed in this paper can use relatively shorter EEG signals and less acquisition channels to obtain better accuracy. Although paper (Soriano et al., 2017) needs the shortest signals, the number of channels is the most and the examination of MEG is more complicated than EEG.

**Table 5:** The epileptic detection accuracy and EEG signal length of this paper and other studies.

**Method**	**Accuracy %**	**Signal length**	**The number of channels**	**Type of signal**
This paper	88.30	20 s	16	EEG
Krishnan et al. (2014)	87	2 h	6	EEG
De Lathauwer et al. (2000)	89.01	20 min	32	EEG
Soriano et al. (2017)	86	5 s	102	MEG

## 5. Conclusion

This study proposes a feature tensor-based detection model to detect epilepsy by using short-time interictal EEG data. This method tends to construct directed binary brain networks based on different basic EEG rhythms and to use the edge removal method to analyze topological features for constructing multi-way tensors. Besides, this study uses Tucker decomposition for obtaining core tensors to distinguish epileptic patients from healthy controls. The experimental results show that the specificity, sensitivity, and accuracy values of the proposed method are all more than 86% with only 20-s EEG data.

In this paper, in-degree and out-degree are employed to measure the nodes' characteristics in constructed brain networks only the local characteristics of the network are considered in this way. when encountering special nodes like “bridge nodes,”the global characteristics also need to be considered, or there will be some misalignment of the evaluation. in future research, more comprehensive methods will be considered, such as the K-order propagation number approach (Tang et al., 2020). Furthermore, (Liu et al., 2015) introduce the concept of time delay stability (TDS) to quantify coordinated bursts in the activity of brain waves, which reveals information about the dynamic interactions between various brain rhythms at different brain locations. In further research, not only specific brain rhythms or bilateral information should be taken into consideration, but also both the global and local communications.

## Data Availability Statement

The data analyzed in this study is subject to the following licenses/restrictions: The datasets for this study are all the clinical data from the Neurology Department of the General Hospital of Eastern Theater Command. Requests to access these datasets should be directed to Jiafei Dai, 37045613@qq.com.

## Ethics Statement

The studies involving human participants were reviewed and approved by the Ethics Committee of the Eastern Theater General Hospital. The patients/participants provided their written informed consent to participate in this study.

## Author Contributions

CS planed, wrote, and revised the whole manuscript. YH prepared the figures and the original model. JM searched the bibliography and proofread the manuscript. JD provided the clinical data. LH supervised the whole project and offer constructive guidance. All authors contributed to the article and approved the submitted version.

## Conflict of Interest

The authors declare that the research was conducted in the absence of any commercial or financial relationships that could be construed as a potential conflict of interest.
